# Applications of Antioxidant Nanoparticles, 2nd Edition

**DOI:** 10.3390/antiox15030340

**Published:** 2026-03-09

**Authors:** Rita Cortesi, Maddalena Sguizzato

**Affiliations:** Department of Chemical, Pharmaceutical and Agricultural Sciences (DoCPAS), University of Ferrara, 44100 Ferrara, Italy; sgzmdl@unife.it

It is known that the overproduction of reactive oxygen species (ROS), which regulate numerous physiological processes, can generate oxidative stress, responsible for the onset of several pathologies [1]. Therefore, a useful therapeutic approach to reducing or neutralizing oxidative stress is the administration of antioxidants. This has been addressed in several fields (i.e., pharmaceuticals, cosmetics, and food) with different types of formulations, including the use of nanoparticles, which have transformed the field of antioxidant therapeutics through enabling specialized nano-delivery systems. In our previous Special Issue “Applications of Antioxidant Nanoparticles”, we summarized and published recent advances in the design, production, characterization, use, and effects of antioxidant nanoparticles.

In this Special Issue of *Antioxidants* entitled “Applications of Antioxidant Nanoparticles, 2nd Edition”, we continue to compile the latest breakthroughs in the design, production, characterization, and utilization of antioxidant nanoparticles in the biomedical, pharmaceutical, cosmetic, and food sectors. The merging of antioxidant therapy and nanomedicine marks a major advancement in the treatment of oxidative disorders, offering more accurate, efficient, and biocompatible approaches.

In this second edition, seven research articles and three reviews have been collected. Specifically, the seven research articles can be divided into two main groups depending on the organic (contributions 1, 2, and 5) or inorganic (contributions 3, 4, 6, and 7) nature of the studied nanoparticle system.

In drug delivery, organic nanoparticles are biocompatible carriers encapsulating therapeutic agents able to improve solubility, protect against degradation, and enable targeted and/or sustained release. They enhance efficacy in treating diseases like cancer by reducing toxicity and promoting controlled, site-specific drug accumulation [2,3,4,5].

Among the contributions of the first group, the research by Baldassarre and colleagues (contribution 1) evaluates the influence of some excipients on the performance of nanoparticle formulations. Indeed, the effect of two different nonionic surfactants, polyvinyl alcohol (PVA) and Tween 20, on the production of organic polymer-based nanoparticles (i.e., polylactic-co-glycolic acid) is described and a thorough discussion is given, in which potential applications in skincare are proposed. Different parameters were investigated in this research, and the results revealed that the type of surfactant affects the stability of nanoparticles and their retention and cellular uptake, neutralizing oxidative stress in skin cells. Contributions 2 and 5 investigate the organic polymer chitosan as a good candidate for the potential treatment of human pathologies, such as nonalcoholic fatty liver disease (NAFLD) and breast cancer. Notably, Alfawaz and colleagues propose loaded chitosan nanoparticles for enhanced bioavailability, solubility, and sustained release, and support their potential as an advanced nanotherapeutic strategy for NAFLD management, specifically acting on inflammation. Similarly, Aarthi Jeganathan’s group demonstrate that loaded chitosan nanoparticles enable the sustained release of active compounds, supporting improvements in various biochemical, enzymatic, and histopathological parameters relating to breast cancer pathology, such as tumor volume and lipid peroxidation.

The remaining contributions (3, 4, 6, and 7) describe the employment of inorganic nanoparticles. Compared to organic materials, inorganic nanoparticles are versatile, stable, and biocompatible drug delivery vehicles; therefore, they are proposed for different purposes, such as targeted therapy, gene delivery, and imaging. Key types include gold, silica, iron oxide, and ceramic, offering advantages like controlled release, high payload capacity, and protection against enzymatic degradation [6].

In fact, inorganic nanoparticles exhibit unique physicochemical properties that allow them to be functionalized with various specific ligands to enhance their affinity for target cells or molecules, making them particularly effective in cancer treatment through improving tumor imaging and drug penetration [7,8,9,10]. In this context, Mao and colleagues (contribution 3) demonstrate that selenium supplementation mitigates the damage caused by H_2_O_2_ to the intestinal morphology of mice jejunum and restores the level of related inflammatory factors. Along these lines, Gutierrez-Albanches’ group (contribution 4) investigated the potential of silver nanoparticles to deliver *Pseudomonas shirazensis* metabolites, with the aim of enhancing the bioactivity of rosemary extracts in postharvest applications. Moreover, the use of magnetite nanoparticles is proposed by Ilie and colleagues (contribution 6) as a drug delivery system for the dual encapsulation of bioactive compounds to treat the gut microbiota dysbiosis that occurs in cancer therapy, demonstrating synergistic antioxidant and antibacterial effects. In addition, the zinc-containing bioactive glass nanoparticles designed and developed by Zhu’s group (contribution 7) are proposed to improve antioxidant and anti-inflammatory effects, facilitating bone and tissue regeneration through exploiting free radical scavenging activity and immunomodulation.

Finally, the reviews by Shi and Hallan’s groups (contribution 8 and 9) offer an overview of the applications of nanoparticle systems in the treatment of autoimmune and lung diseases. Shi and colleagues specifically addressed immune-mediated inflammatory diseases, such as alopecia areata and multiple sclerosis, in which oxidative stress plays a key role in onset and progression, and highlighted the potential of nano-antioxidants as a promising therapeutic approach, primarily due to their stability and their capability for targeted delivery (contribution 8). Hallan’s review, on the other hand, considers the use of organic nanostructures such as cyclodextrins in the treatment of oxidative stress-driven lung diseases such as cystic fibrosis, pulmonary hypertension, chronic airway diseases, and lung cancer (contribution 9). The third review offers an overview of the nanoformulation of a natural compound, alpha-lipoic acid, with potent antioxidant properties that protect cells and tissues from oxidative stress. Specifically, it describes the engineering of the molecule of interest as a novel, innovative formulation strategy, which promises to be an effective drug delivery system (contribution 10).

Overall, all of the reviews emphasize that nanoparticulate systems can overcome the problems of poor solubility, stability in aqueous solutions, and bioavailability after administration, which hinder their clinical utility. However, in some cases, the lack of physiological models for assessing the efficacy of tissue-oriented drug targeting and subsequent clinical studies remains an open issue.

In summary, through this second edition, we have attempted to expand the awareness of researchers regarding the different applications of nanoparticles in counteracting or regulating the effect of oxidative stress in different diseases, either through topical or systemic treatments. The combination of green production methods and natural molecules has also been explored in this second edition, demonstrating multifunctional hybrid approaches that mitigate oxidative stress, inflammation, and microbial potential. Finally, the studies collected in this Special Issue emphasize the potential of nanotechnological systems in modulating the activity of the molecules being carried—such as antioxidant actives—after administration, enhancing their therapeutic activity, stability, and targeted uptake and delivery across different biomedical applications characterized by redox environments.
